# Neuromuscular Training Improves Lower Extremity Biomechanics Associated with Knee Injury during Landing in 11–13 Year Old Female Netball Athletes: A Randomized Control Study

**DOI:** 10.3389/fphys.2017.00883

**Published:** 2017-11-07

**Authors:** Amanda J. Hopper, Erin E. Haff, Christopher Joyce, Rhodri S. Lloyd, G. Gregory Haff

**Affiliations:** ^1^Centre for Exercise and Sports Science Research, Edith Cowan University, Joondalup, WA, Australia; ^2^School of Health Sciences, The University of Notre Dame Australia, Fremantle, WA, Australia; ^3^Youth Physical Development Centre, Cardiff School of Sport, Cardiff Metropolitan University, Cardiff, United Kingdom; ^4^Sport Performance Research Institute New Zealand, Aukland University of Technology, Aukland, New Zealand; ^5^Centre of Sport Science and Human Performance, Waikato Institute of Technlogy, Hamilton, New Zealand

**Keywords:** female, injury prevention, landing mechanics, strength training, youth

## Abstract

The purpose of this study was to examine the effects of a neuromuscular training (NMT) program on lower-extremity biomechanics in youth female netball athletes. The hypothesis was that significant improvements would be found in landing biomechanics of the lower-extremities, commonly associated with anterior cruciate ligament (ACL) injury, following NMT. Twenty-three athletes (age = 12.2 ± 0.9 years; height = 1.63 ± 0.08 m; mass = 51.8 ± 8.5 kg) completed two testing sessions separated by 7-weeks and were randomly assigned to either a experimental or control group. Thirteen athletes underwent 6-weeks of NMT, while the remaining 10 served as controls and continued their regular netball training. Three-dimensional lower-extremity kinematics and vertical ground reaction force (VGRF) were measured during two landing tasks, a drop vertical jump and a double leg broad jump with a single leg landing. The experimental group significantly increased bilateral knee marker distance during the bilateral landing task at maximum knee-flexion range of motion. Knee internal rotation angle during the unilateral landing task at maximum knee flexion-extension range of motion was significantly reduced (*p* ≤ 0.05, *g* > 1.00). The experimental group showed large, significant decreases in peak vertical ground reaction force in both landing tasks (*p* ≤ 0.05, *g* > −1.30). Control participants did not demonstrate any significant pre-to-post-test changes in response to the 6-week study period. Results of the study affirm the hypothesis that a 6-week NMT program can enhance landing biomechanics associated with ACL injury in 11–13 year old female netball athletes.

## Introduction

Adolescent female athletes experience non-contact anterior cruciate ligament (ACL) injuries at a 4–6 times higher rate than males participating in the same sports (Hewett et al., 1999, 2006). One possible explanation related to this observation is the absence of the neuromuscular spurt following maturation in female athletes, which results in neuromuscular imbalances in muscle strength and activation patterns (Myer et al., 2004). These imbalances increase loading on the joint consequently increasing non-contact ACL injury risk. Appropriately designed and implemented neuromuscular training (NMT) programs have been shown to decrease ACL injury risk in adolescent female athletes by improving their neuromuscular control and dynamic knee stability (Myer et al., 2005; Chappell and Limpisvasti, 2008). It appears that NMT programs inclusive of both resistance and plyometric training are most effective at reducing non-contact ACL injury risk in female athletes under the age of 18 (Yoo et al., 2010). These programs focus on improving neuromuscular strength and control, proprioception, motor control, fundamental movement patterns and functional biomechanics, with the aim of decreasing ACL injury risk (Myer et al., 2005).

The effectiveness of NMT programs to decrease ACL injury risk and improve lower extremity biomechanics has been studied within adolescent soccer, basketball and volleyball athletes (Myer et al., 2005; Chappell and Limpisvasti, 2008) as these sports exhibit high ACL injury rates (Boden et al., 2000). One sport that has not received a lot of attention in the scientific literature examining NMT and knee injury risk management is the sport of netball. Netball also displays high lower extremity injury incidence rates, with ACL rupture being the most commonly occurring injury in the sport (McManus et al., 2006). In non-professional netball athletes, over a 2 year period ligament sprains or tears accounted for 61.2% of total sports related injuries (Finch et al., 2002). Similarly, over a 3-day state netball competition there were 139.4 injuries per 1,000 players, with the knee and ankle being the most frequently injured body part, and ligament sprains being the most common injury (Hume and Steele, 2000). Furthermore, 65% of these injuries came from the under 17's squad which was the youngest division in the competition. Based upon these statistics it is apparent that there is a need for the implementation of effective NMT programs that specifically target the prevention of ACL injuries in netball athletes of all ages.

It has been suggested that the primary cause of lower limb injury in netball is incorrect landing technique (Hopper et al., 1995), combined with rapid deceleration or an abrupt landing (Cowling and Steele, 2001). In netball, a key rule requires players to only take a maximum of one and half steps whilst in possession of the ball, therefore the player must rapidly decelerate and stop after receiving the ball in order not to violate this rule (Steele, 1990). Jumping, landing, leaping and lunging movements are commonly performed during both netball practice and competition. For example, Lavipour (2011) found that on average netball players will perform one jump per minute during a game with athletes landing unilaterally 67% of the time, due to most of their jumps requiring them to jump to receive the ball. The impact of the frequency of jump landings on overall injury risk is clearly seen in the work of Stuelcken et al. (2015), where out of 16 ACL injuries in elite netball players, 13 of these occurred with the player landing from a jump.

Mechanisms of ACL injury in female athletes have been extensively studied and reported in scientific literature (Boden et al., 2000, 2010; Hewett et al., 2004, 2006; Hewett, 2005). Anterior cruciate ligament injury occurs during dynamic lower extremity valgus, which typically involves a combination of hip adduction and internal rotation, knee abduction, tibial external rotation and anterior translation, and ankle eversion (Hewett et al., 2006). The governing mechanisms for ACL injuries in young female athletes is generally associated with poor neuromuscular control leading to altered lower limb biomechanics such as; increased knee valgus and foot pronation angles, and decreased hip and knee flexion, and hip abduction during cutting and landing (Hewett et al., 2006). In particular, these risk factors are exacerbated as they mature and peak following the post-pubertal stage of development (Myer et al., 2009). Prior to puberty there is no difference in lower limb biomechanics during landing between males and females (Jackson et al., 2010). However, following puberty, females demonstrate changes in hip and knee biomechanics that predispose them to ACL injury (Hewett et al., 2004). In elite netball, Stuelcken et al. (2015) reported valgus knee collapse in 12 of 13 ACL injuries. One possible explanation for this occurrence may be related to an increase in joint laxity and reduction in lower extremity control that maturing athletes commonly experience. The consequence of this occurrence is a decrease in passive joint stability that may increase the tendency toward valgus knee motions (Quatman et al., 2008; Myer et al., 2009).

Previous studies have shown that with maturity (pre-pubertal to post-pubertal) female athletes land with greater knee extension, and greater extension moments and powers (Decker et al., 2003; Hass et al., 2003) thereby increasing the amount of force directly placed upon the ACL (Renström et al., 1986). Boden et al. (2000) found of athletes who had sustained an ACL injury the majority of them reported the knee being close to full extension at the time of injury. Another possible factor that can exacerbate this problem is weakness of the hip musculature (Powers, 2010). If the hip musculature is weak the hip can move into adduction during loading, which can place the knee into a valgus position (Jacobs et al., 2007; Powers, 2010) increasing the overall risk of sustaining an ACL injury. This problem is particularly evident in female athletes who demonstrate significantly more hip adduction and hip internal rotation during weight bearing activities than their male counterparts (Jacobs et al., 2007).

Neuromuscular training has been shown to decrease potential for ACL injury by improving biomechanical deficiencies typically associated with injury (Hewett et al., 1999; Myer et al., 2005). Myer et al. (2013) indicated that there is a potential window of opportunity to decrease ACL injury risk in young female athletes if NMT is implemented prior to the onset of puberty. A meta-analysis by Yoo et al. (2010) found NMT incorporating both plyometric and strength training were most effective in preventing ACL injuries in female soccer, handball and basketball athletes, specifically those under the age of 18. In support of this contention Myer et al. (2005) found following a 6-week NMT intervention inclusive of strength training, plyometric and balance training, 14–16 year old female basketball, volleyball and soccer athletes were able to significantly increase their knee flexion angle and decrease internal knee valgus in response to a drop vertical jump task (DVJ). The effects of NMT to prevent incidence of ACL injury has been extensively studied in adolescent basketball, soccer and volleyball athletes (Hewett et al., 1999; Myer et al., 2005; Pfile et al., 2013). Hewett et al. (1999) found untrained high school female athletes presented a significantly higher incidence of sports related knee injury then the male control group, however female athletes who had participated in a NMT program showed no significant difference in injury incidence in contrast to the male control group. However, the effects of a 6-week NMT intervention in preventing factors associated with ACL injury is yet to be quantified with netball athletes.

Therefore, the aim of this study is to determine the effects of a 6-week NMT program on lower extremity biomechanics in youth female netball athletes. The hypothesis is that significant improvements will be seen in the biomechanical measures commonly related to ACL injury in female athletes (increased range of motion [ROM] in flexion-extension, decreased abduction-adduction and internal-external rotation, and decreased ground reaction force) following the training intervention.

## Materials and methods

**Figure 1:**
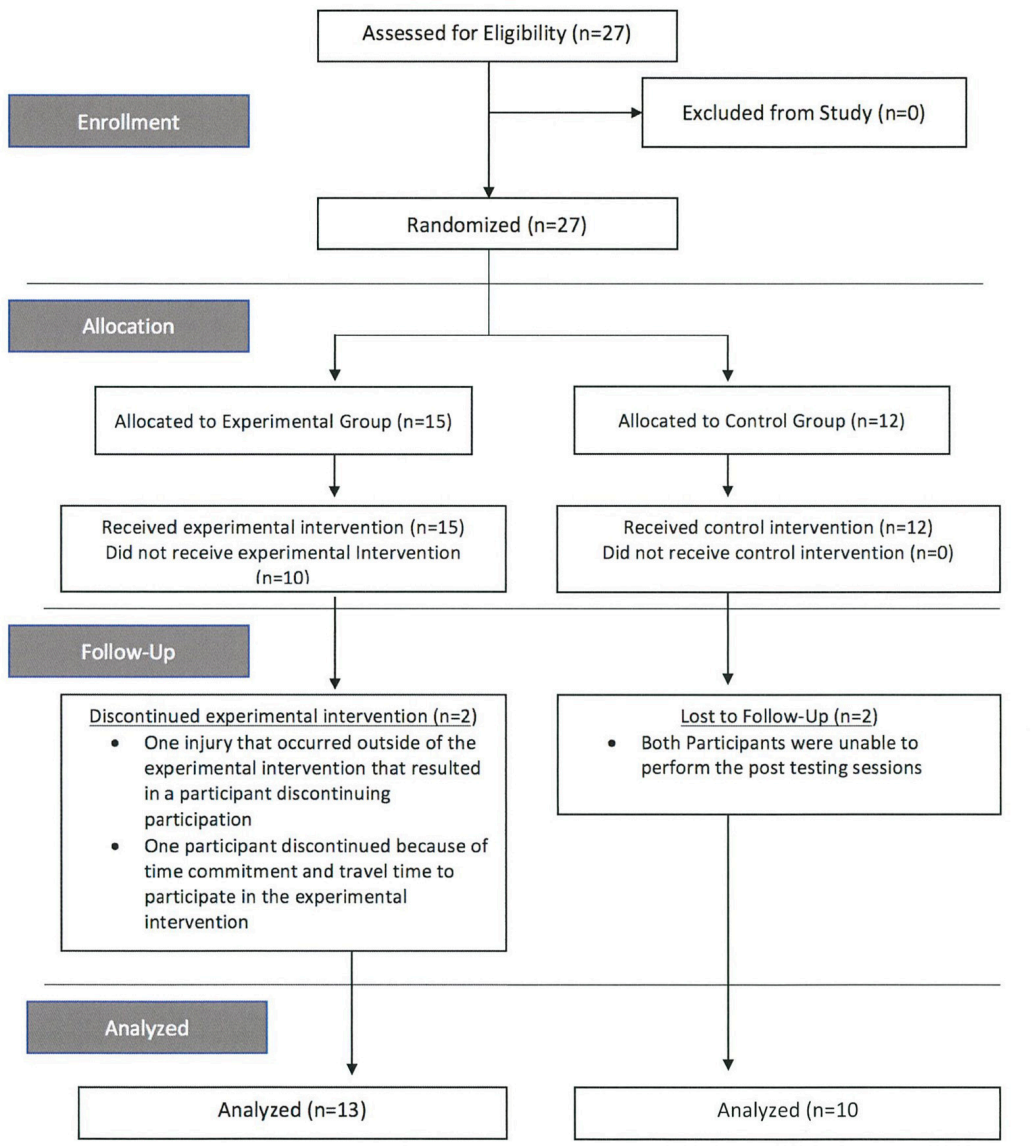
Subject Recruitment and Randomization Process.

Twenty-three youth female netball athletes (age = 12.2 ± 0.9 yrs; height = 1.63 ± 0.08 m; mass = 51.8 ± 8.5 kg) were recruited from local netball clubs to participate in the study. The sample size was chosen based upon a power analysis (β = 0.829) indicating a minimum of 16 athletes are needed for the study. Further, samples size was considered based on similar multiple training group studies involving youth participants (Faigenbaum et al., 2007; Chaouachi et al., 2014; Meylan et al., 2014) who used sizes of *n* ≤ 12, *n* ≤ 14, and *n* ≤ 17 respectively. Participants verbally reported no previous history of lower limb injury and no previous experience partaking in NMT. All participants were instructed to maintain their normal netball training regime. Participants were recruited for participation in this randomized control trial study and divided into either an experimental (EG; *n* = 13) or control group (CG; *n* = 10) in a blinded fashion following the first battery of testing (Figure 1). Control participants were instructed not to partake in any resistance training activity during the course of the study. No significant differences in age, height and mass were found between the EG and CG in either the pre- (Height: EG = 1.64 ± 0.07 m; CG = 1.64 ± 0.10 m; Mass: EG = 50.7 ± 8.8 kg; CG = 53.3 ± 8.2 kg) or post-testing (Height: EG = 1.63 ± 0.07 m; CG = 1.63 ± 0.10 m; Mass: EG = 52.0 ± 8.9 kg; CG = 53.9 ± 8.4 kg) sessions (*p* ≥ 0.05). All participants had participated in competitive netball for more than 4 years. The Human Research Ethics Committee at Edith Cowan University approved all test procedures and written informed parental consent and participant assent was obtained prior to commencement of the study in accordance with the Declaration of Helsinki. Participants completed the Physical Activity Readiness Questionnaire (PAR-Q) prior to beginning of the study and a modified Pubertal Maturation Observational Scale (PMOS) (Davies and Rose, 2000) was used to classify participants into maturational categories: pre-pubertal mid-pubertal and post-pubertal. There was no significant difference in maturational categories (*p* = 0.051) found between the experimental (2.2 ± 0.9) and control groups (2.8 ± 0.4) indicating that both groups are primarily made up of pubertal athletes.

The present investigation explored the addition of NMT to traditional youth netball training (i.e., ~1–2 court based netball training sessions and ~1–2 netball games per week) on landing biomechanics in youth netball athletes. All participants completed a 1-week familiarization program during 3 × 60 min training sessions comprised of back squats, push ups, static lunges, horizontal pull ups and glute bridges prior to the initiation of any testing. Baseline testing occurred following the familiarization program, 1 week before the initial NMT session. Post-testing was performed approximately 7 weeks after baseline testing on the EG and CG. The 6-week NMT intervention involved 3 × 60 min training sessions per week performed on non-consecutive days, for a total of 18 training sessions. A minimum of 48 h separated each resistance training session to allow for sufficient recovery. The CG participated in both baseline- and post-testing sessions however did not receive any NMT during the study period.

A 10 camera MX-F20 Vicon-Peak Motion Analysis system (Oxford Metrics Ltd., Oxford, UK) operating at 250 Hz was used to capture hip and knee kinematics using the validated Vicon Plug-In Gait model, with each participant fitted with 37 retro-reflective markers 14 mm in diameter. A static trial was collected to align the joint coordinate system to the laboratory. Vertical ground reaction force data were collected using a 600 × 900 mm triaxial force platform (Kistler, Type 9287CA, Switzerland) recorded at 1,000 Hz with Vicon Nexus Software (ver. 1.6.1, Oxford Metrics Ltd., Oxford, UK).

Participants were instructed to perform a drop vertical jump (DVJ) task and a broad jump with a single leg landing (SL). The DVJ trials commenced with each participant standing on a box 31 cm in height with their feet positioned 35 cm apart. Each participant was instructed to step off the box onto the force platform with their dominant leg and immediately perform a maximal vertical jump (Markwick et al., 2015). During the SL task, participants were instructed to perform a maximal forward jump from two legs and land on the force plate with their dominant leg, which was determined by asking them which leg they would use to kick a ball (Ford et al., 2003). Participants were given three practice trials of each jump prior to data collection to ensure the markers were secure, and to confirm they understood the movement pattern.

The training program utilized in the present study was a synthesis of previously published ACL injury prevention studies (Myer et al., 2005, 2013; Yoo et al., 2010). The components of the integrated NMT program employed in this study included a combination of plyometric and strength training. All sessions were conducted by accredited strength and conditioning coaches (Australian Strength and Conditioning Association) and a certified strength and conditioning specialist (National Strength and Conditioning Assosiation). The dynamic warm-up remained consistent throughout all sessions over the 6-week training period, whilst the plyometric and strength exercises were split into two 3-week blocks (Tables 1, 2). Training intensity, volume and complexity of exercise increased in the second training block, with movement quality and technical competency being prioritized at all times. Participants recorded load lifted on each exercise for each set throughout the training intervention. This was then used to determine load for following week in combination with the OMNI Rate of Perceived Exhaustion (RPE) scale (Robertson et al., 2008) which was used to set a goal RPE for the session. All participants were familiarized with the use of the RPE scale prior to the first session and instructed to use the pictures and the words to provide a rating. The RPE goal incrementally increased each week with the first week being relatively easy (3–4 rating), second week being somewhat hard (5–6 rating) and the third week being hard (7–8 rating). This RPE guide was repeated for the second training block. Participants were also asked after each set to rate their RPE to determine if load could be increased, in conjunction with the opinion of the strength and conditioning coach to ensure technical efficiency and safety at all times.

Table 1Neuromuscular Training Program for Weeks 1 through 3 for the experimental group.

**Table:** 

**Exercises**	**Sets**	**Reps**	**Rest**
	**Dynamic Warm-up**		
	**Sessions #1, #2, and #3**		
High Knee March	2	10	60
Sumo Squat Arms MB Overhead	2	10	60
Arm Swing and Lunge	2	10	60
Backward Hip Flexion Walk	2	10	60
Superman	2	10	60
Knee Grab to Lunge and Twist	2	10	60
Carioca	2	10	60
Rocket Jump	2	10	60
	**Plyometric Training**		
	**Sessions #1, #2, and #3**		
½ Squat Jump	3	5	90
Lateral Bound with Stick	3	5	90
Single Leg Push Off	3	5	90
90° Spin Jump	3	5	90

**Table:** 

**Strength Training**							
**Sessions #1 and #3**				**Session #2**			
**Exercises**	**Sets**	**Reps**	**Rest**	**Exercises**	**Sets**	**Reps**	**Rest**
Back Squat	3	5	90	Front Squat	3	5	90
Bench Press	3	5	60	Incline Press	3	5	60
Medicine Ball Static Lunge (bent arms)	3	5	60	Medicine Ball Static Lunge (straight arms)	3	5	60
Military Press	3	5	60	Behind Neck Press	3	5	60
Horizontal Pull Up	3	5	60	Horizontal Pull Up	3	5	60

*Ratings of perceived exertion was used to guide intensity*.

Table 2Neuromuscular Training Program for Weeks 4 through 6 for the experimental group.

**Table:** 

**Exercises**	**Sets**	**Reps**	**Rest**
	**Dynamic Warm-up**		
	**Sessions #1, #2, and #3**		
High Knee March	2	10	60
Sumo Squat Arms MB Overhead	2	10	60
Arm Swing and Lunge	2	10	60
Backward Hip Flexion Walk	2	10	60
Superman	2	10	60
Knee Grab to Lunge and Twist	2	10	60
Carioca	2	10	60
Rocket Jump	2	10	60
	**Plyometric Training**		
	**Sessions #1, #2, and #3**		
Medicine Ball ½ Squat Jump	3	5	90
Medicine Ball Lateral Bound	3	5	90
Single Leg Push off (Med Box)	3	5	90
180° Spin Jump	3	5	90

**Table:** 

**Strength Training**							
**Sessions #1 and #3**				**Session #2**			
**Exercises**	**Sets**	**Reps**	**Rest**	**Exercises**	**Sets**	**Reps**	**Rest**
Back Squat	3	8	90	Front Squat	3	8	90
Incline Bench Press	3	8	60	Bench Press	3	8	60
Split Squat –(Back foot elevated)	3	8	60	Split Squat –(Front foot elevated)	3	8	60
Chin-up^*^	3	8	60	Bent Over Row	3	8	60
Romanian Deadlift	3	8	60	Backward Alternating Lunge	3	8	60

*Ratings of perceived exertion was used to guide intensity*.

* *If athletes could not perform a chin-up, resistance bands were used to assist them. In this case the resistance bands were modified to reduce reliance on the band across the training period*.

### Knee and hip kinematics

The Vicon plug-in gait model was used to obtain hip and knee kinematics. Coordinate data from each of the three-dimensional marker trajectories were filtered using a low-pass Butterworth filter at a cut off frequency of 12 Hz. Knee joint biomechanics; flexion-extension, abduction-adduction, and external-internal rotation angles, and hip joint biomechanics; flexion-extension, abduction-adduction and external-internal rotation angles at initial contact (i.e., intitial contact after stepping of the box) and maximum knee flexion-extension ROM were calculated from the embedded joint coordinate system. Three successful trials were collected for the DVJ task and the SL task and an average of the three trials for each landing task was used for data analysis. Positive values indicated knee flexion, knee abduction, knee external rotation, hip flexion, hip abduction and hip external rotation. Negative values represented knee adduction, knee internal rotation, hip adduction and hip internal rotation.

### Bi-lateral knee valgus

Bi-lateral knee valgus (abduction) was calculated from the frontal plane by exporting the coordinate data as a text file in Microsoft Excel and calculating the distance (meters) between the right and left lateral knee markers at the point before initial contact (PIC) and maximum knee flexion-extension ROM as described in Ford et al. (2003).

### Ground reaction force and flight time

Vertical ground reaction force (VGRF) data were derived from the embedded force platform through Vicon Nexus software. During the DVJ task, peak VGRF was recorded immediately following the box drop and immediately following the vertical jump. Peak VGRF was recorded following the jump in the SL landing task. Flight time was determined from the Kistler force plate system.

All results are represented as means ± SD. To compare differences between the pre- and post-test values for the experimental and control groups a 2 × 2 (group × time) repeated measures ANOVA was used. A significance level of *p* ≤ 0.05 was set for all statistical analyses. If significant *F*-values were found, paired comparisons were used to determine differences in conjunction with a Holm's sequential Bonferonni correction to account for Type I errors. Homogeneity of variance was tested with the use of a Kolmogorov-Smirnov test and liliefors correction. If homogeneity of variance was violated the Kruskal-Wallis H test was used to determine statistical significance. One-way ANOVAs were performed on all variables to determine raw difference scores (post-pre) between groups. Effect sizes were calculated as Hedges *g* and were interpreted as the following: trivial, < 0.2; small, 0.2–0.5; medium, 0.5–0.8; large, 0.8–1.3; and very large, >1.3 (Hopkins, 2010). Intra-class correlation coefficients (ICCα) were calculated for all variables to determine within-trial reliability. Analysis of scores in the present study demonstrated high reliability across all variables for the DVJ and SL landing tasks as indicated by *ICC*α ≥ 0.80 (Atkinson and Nevill, 1998). All statistical analyses were conducted using SPSS (SPSS 23.0.0.0, SPSS Inc., Chicago, IL).

## Results

### Hip and knee kinematics

Significant group x time interactions were noted when examining the knee flexion-extension angle (*p* = 0.009; *g* = 1.20) and knee external rotation (*p* = 0.043, *g* = 0.91) at intitial contact during the DVJ task. Additionally, a group × time interaction was noted for hip abduction angle at maximum knee flexion-extension ROM (*p* = 0.020; *g* = 1.05). Baseline and post-testing lower extremity kinematics for the DVJ task are presented in Table 3.

**Table 3:** Drop vertical jump task biomechanics during pre- and post-intervention testing.

**Variable**			**Initial contact**		**Maximum knee flexion angle**	
			**Experimental**	**Control**	**Experimental**	**Control**
			**Mean ± SD**	**Mean ± SD**	**Mean ± SD**	**Mean ± SD**
Knee Flexion	Pre	(°)	34.4 ± 5.3	36.2 ± 3.3	106.9 ± 8.9	103.7 ± 3.2
	Post	(°)	37.1 ± 4.8^*^	31.3 ± 6.5	114.8 ± 10.1	102.5 ± 7.2
	Δ (Post-Pre)	(°)	2.7 ± 5.4^*^	−4.9 ± 5.9	7.9 ± 12.2	−1.3 ± 9.2
Knee Abduction	Pre	(°)	−4.1 ± 5.8	−10.0 ± 5.5	−13.1 ± 4.5	−20.7 ± 3.4
	Post	(°)	2.6 ± 5.4	−2.6 ± 8.8	0.66 ± 9.3	−13.4 ± 2.2
	Δ (Post-Pre)	(°)	6.7 ± 6.3	7.4 ± 11.8	13.7 ± 9.6	7.2 ± 5.2
Knee External Rotation	Pre	(°)	−17.6 ± 10.4	−13.4 ± 12.8	2.3 ± 16.7	−4.8 ± 2.0
	Post	(°)	−9.9 ± 14.2^*^	−21.5 ± 8.7	8.1 ± 13.3	−10.2 ± 15.7
	Δ (Post-Pre)	(°)	7.6 ± 15.3^*^	−8.1 ± 18.7	5.8 ± 19.3	−5.4 ± 16.1
Hip Flexion	Pre	(°)	41.1 ± 4.5	43.7 ± 6.7	94.7 ± 4.9	87.2 ± 11.3
	Post	(°)	46.2 ± 2.7	44.1 ± 10.7	99.2 ± 7.5	88.7 ± 13.3
	Δ (Post-Pre)	(°)	5.0 ± 5.3	0.4 ± 7.8	4.5 ± 8.3	1.5 ± 5.7
Hip Abduction	Pre	(°)	10.4 ± 4.6	8.5 ± 3.4	6.7 ± 5.9	4.4 ± 6.7
	Post	(°)	10.7 ± 4.4	8.7 ± 3.9	13.9 ± 3.1^*^	5.5 ± 7.6
	Δ (Post-Pre)	(°)	0.3 ± 10.7	0.2 ± 3.5	7.3 ± 6.2	1.1 ± 4.5
Hip External Rotation	Pre	(°)	−10.6 ± 11.2	−19.5 ± 5.5	−7.3 ± 12.2	−19.4 ± 8.9
	Post	(°)	−6.3 ± 9.2	−8.5 ± 11.2	2.0 ± 12.4	−14.4 ± 3.8
	Δ (Post-Pre)	(°)	4.4 ± 11.2	11.0 ± 14.8	9.4 ± 13.0	4.9 ± 8.7

* *Indicates statistical significance p ≤ 0.05*.

Follow-up comparisions revealed no significant differences between groups at baseline testing for knee flexion-extension ROM (*p* = 0.373, *g* = −0.39) and knee external rotation (*p* = 0.408, *g* = −0.35) at initial contact. Additionally, there were no significant differences noted between the groups for hip abduction (*p* = 0.308, *g* = −0.44) and hip flexion ROM (*p* = 0.289, g = −0.45) at baseline testing. When examining the between group differences at post-testing there was a significant difference between the EG and the CG for knee-extension flexion ROM (*p* = 0.002; *g* = 1.20) and knee external rotation (*p* = 0.044, *g* = 0.90) at initial contact. There was a significant difference noted between the EG and CG when examining the hip abduction at maximum knee flexion-extension ROM (*p* = 0.002; *g* = 1.05).

When examining the SL broad jump landing task there was a significant group × time interaction for knee flexion (*p* = 0.001, *g* = 1.69) and external rotation (*p* = 0.009, *g* = 0.99) at initial contact were noted between the EG and CG. Additionally, there was a significant group x time interaction for knee external rotation (*p* = 0.023, *g* = 0.73) at maximum knee flexion-extension ROM. When examining the change score between pre and post testing the EG demonstrated a significantly greater knee flexion angle at initial contact (*p* = 0.001; *g* = 2.84) when compared to the CG. Additionally, the EG demonstrated a significantly greater (*p* = 0.009; *g* = 1.21) external rotation of the knee at initial contact. At maximum knee flexion-extension ROM the EG demonstrated a significantly greater (*p* = 0.023; *g* = 1.0) external rotation of the knee when compared to the control group (Table 4).

**Table 4:** Single leg landing task biomechanics during pre- and post-intervention testing.

**Variable**			**Initial contact**		**Maximum knee flexion angle**	
			**Experimental**	**Control**	**Experimental**	**Control**
			**Mean** ± **SD**	**Mean** ± **SD**	**Mean** ± **SD**	**Mean** ± **SD**
Knee Flexion	Pre	(°)	20.3 ± 3.2	23.3 ± 3.0	58.8 ± 6.4	60.2 ± 5.6
	Post	(°)	24.3 ± 2.9^*^	18.5 ± 9.0	65.3 ± 11.7	60.0 ± 12.0
	Δ (Post-Pre)	(°)	4.1 ± 2.8^*^	−4.5 ± 6.8	6.6 ± 7.9	−0.3 ± 10.4
Knee Abduction	Pre	(°)	−5.66 ± 4.14	−8.46 ± 3.85	−7.39 ± 5.63	−12.15 ± 2.97
	Post	(°)	−1.71 ± 3.96	−4.67 ± 6.03	0.35 ± 8.80	−8.94 ± 5.04
	Δ (Post-Pre)	(°)	3.95 ± 4.16	3.79 ± 5.95	7.74 ± 7.42	3.21 ± 5.66
Knee External Rotation	Pre	(°)	−22.95 ± 4.15	−22.80 ± 1.85	−3.96 ± 11.60	−8.19 ± 5.70
	Post	(°)	−7.56 ± 11.47^*^	−21.39 ± 8.76	5.11 ± 14.11^*^	−12.29 ± 6.77
	Δ (Post-Pre)	(°)	15.39 ± 12.42^*^	1.40 ± 8.85	9.08 ± 14.89^*^	−4.09 ± 7.09
Hip Flexion	Pre	(°)	45.72 ± 8.61	53.10 ± 11.99	62.39 ± 8.79	63.21 ± 4.52
	Post	(°)	49.78 ± 7.90	50.17 ± 7.15	69.31 ± 6.59	62.94 ± 11.71
	Δ (Post-Pre)	(°)	4.05 ± 6.69	−2.94 ± 9.72	6.92 ± 7.97	−0.28 ± 12.50
Hip Abduction	Pre	(°)	9.53 ± 4.12	8.76 ± 2.00	1.42 ± 2.73	−1.75 ± 2.50
	Post	(°)	9.86 ± 3.5	9.25 ± 2.59	2.65 ± 5.28	0.54 ± 2.71
	Δ (Post-Pre)	(°)	0.32 ± 4.86	0.07 ± 1.96	1.23 ± 5.40	2.28 ± 4.48
Hip External Rotation	Pre	(°)	−14.24 ± 11.68	−24.15 ± 12.98	−7.72 ± 8.84	−15.39 ± 4.20
	Post	(°)	−7.01 ± 7.53	−13.74 ± 15.64	−3.15 ± 9.46	−12.59 ± 6.01
	Δ (Post-Pre)	(°)	7.23 ± 12.15	10.41 ± 19.53	4.57 ± 10.79	3.59 ± 7.30

* *Indicates statistical significance p ≤ 0.05*.

**Figure 2:**
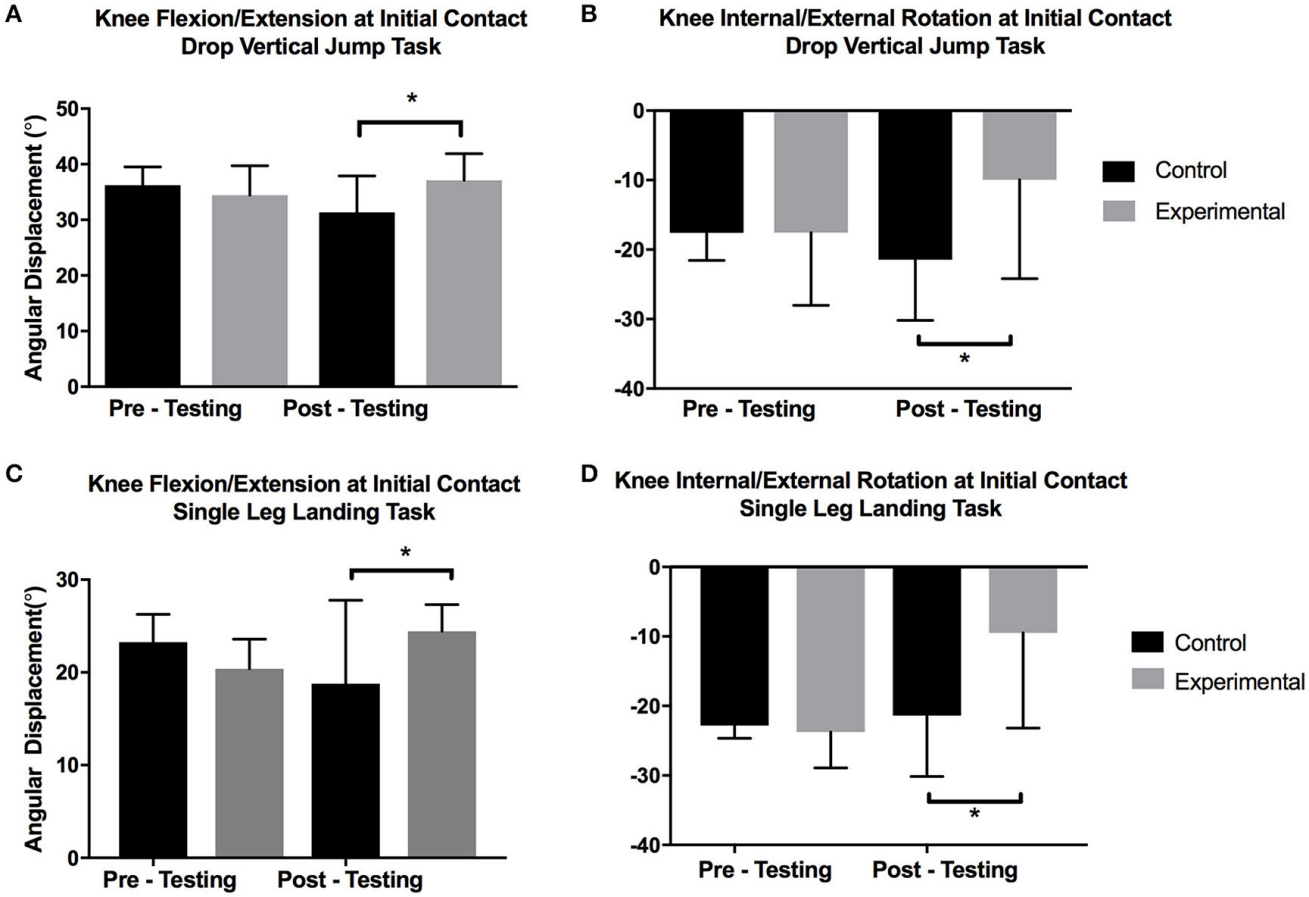
Knee Flexion/Extension and Internal/External Rotations During Jumping Tasks. ^*^Significant difference *p* < 0.05.

When examining the baseline data there were no significant differences between the EG and CG for knee flexion (*p* = 0.051, *g* = −0.89) or external rotation (*p* = 0.606, *g* = −0.22) at initial contact or maximum knee flexion angles (*p* = 0.590, *g* = −0.23) during the SL landing task. However, at the post-testing time point the EG demonstrated significantly greater knee flexion angles at initial contact (*p* = 0.04; *g* = 2.84). Additionally, external rotation of the knee for the EG displayed significantly less external rotation at initial contact (*p* = 0.03; *g* = 1.21) and maximal knee flexion (*p* = 0.003; *g* = 1.02; Figure 2).

### Bilateral knee marker distance

**Figure 3:**
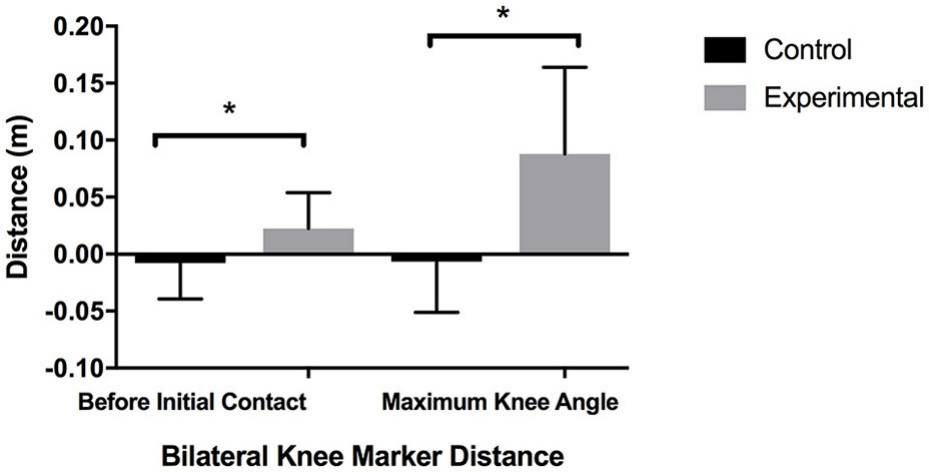
Bilateral Knee Marker Distance. ^*^Significant difference *p* < 0.05.

There was a significant group x time interaction (*p* = 0.045; *g* = 0.92) when examining the distance between the right and left lateral knee markers before initial contact (PIC). Additionally, there was a significant group × time interaction (*p* = 0.003; *g* = 1.45) when examining the distance between the right and left lateral knee markers at maximum knee flexion. Follow-up tests revealed that there were no significant differences between the PIC at pre-testing at the initial contact (*p* = 0.78; *g* = 0.12) and max knee flexion (*p* = 0.800; *g* = 0.02). There was however a significant difference (*p* = 0.004; *g* = 1.43) between the PIC at maximum knee flexion with the experimental group displaying a significantly decreased knee abduction in comparison to CG (Figure 3).

### Ground reaction force and flight time

**Figure 4:**
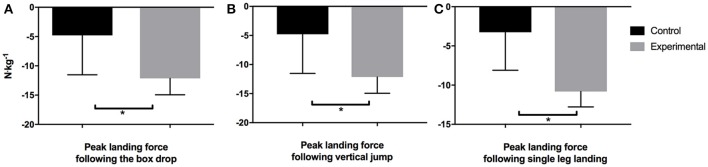
Peak landing forces after a **(A)** Box Drop, **(B)** Vertical Jump, and **(C)** Single Leg Landing. ^*^Significant difference *p* < 0.05.

Ground reaction force (GRF) mean ± SD and effect sizes for the experimental and control conditions for both the DVJ and SL jump tasks during the pre- and post-testing sessions are presented in Table 5. There were no differences between the groups for peak GRF prior to the training intervention in both landing tasks (DVJ: *p* = 0.433, *g* = −0.35; SL: *p* = 0.737, *g* = 0.15). When examining the post-testing DVJ performance the EG demonstrated a significantly (*p* = 0.005, *g* = −1.92) lower peak GRF when compared to the CG. Similarly the EG demonstrated a significantly (*p* = 0.03, *g* = −1.05) GRF during the landing phase following the vertical jump. Furthermore, the EG also showed very large statistically significant decreases in peak GRF during the SL landing task (*p* = 0.001; *g* = −1.74; Figure 4).

**Table 5:** Ground reaction force data for both landing tasks during pre- and post-intervention testing.

**Variable**			**Experimental**	**Control**
			**Mean ± SD**	**Mean ± SD**
Drop Vertical Jump Following Drop	Pre	(N)	1, 162.8|197.3	1, 257.8|278.2
	Post	(N)	546.8|107.5^*^	934.8|277.5
	Δ (Post-Pre)	(N)	−616.1|131.5^*^	−313.0|482.6
Following Vertical Jump	Pre	(N)	1, 221.5|331.7	1, 344.4|234.2
	Post	(N)	611.1|131.5^*^	1, 064.2|364.0
	Δ (Post-Pre)	(N)	−610.6|221.7^*^	−280.2|393.5
Single Leg Landing Task	Pre	(N)	1, 082.3|107.2	1, 061.9|167.7
	Post	(N)	556.8|42.1^*^	877.45|272.2
	Δ (Post-Pre)	(N)	−525.5|95.5^*^	−184.5|266.6

* *Indicates statistical significance p ≤ 0.05*.

Flight time during the DVJ revealed no significant group × time interaction (*p* > 0.05). When raw-difference scores were examined the EG displayed an increase in their flight time (+0.03 ± 0.05 s) whilst the CG displayed a decrease in their flight time (−0.03 ± 0.07 s) however although these differences were not statistically significant (*p* > 0.05) they did display a large effect size (*g* = 0.98).

## Discussion

The objective of the current study was to determine if a comprehensive NMT program inclusive of plyometric and strength training could improve lower extremity biomechanics in 11–13 year old netball athletes. Results of the current study indicated EG demonstrated significant improvements in knee- and hip-joint biomechanics and decreases in peak GRF in both a DVJ and SL landing tasks in comparison to the CG.

The results of the current study support the work of Myer et al. (2005) who used a NMT program comprised of resistance training, plyometric and balance training to improve dynamic knee stability in comparison to an untrained control group. They demonstrated that 14–16 year old female athletes who underwent a NMT intervention were able to significantly improve their knee flexion angle during the landing phase of a box drop. These results are similar to those found in the current study with trained youth netball athletes. However, it should be noted that the current study used a slightly younger population aged 11–13 years. Results indicated that the strength trained youth netball athletes were able to significantly increase their knee flexion angle at initial contact in the DVJ task and SL landing task whilst the control group showed a decrease in knee flexion angle following the intervention. While the EG increases in knee flexion-extension ROM at maximal knee angle during the DVJ were not statistically different from the CG a medium effect size was determined suggesting a potential meaningful effect. Thus, practitioners should realize the biomechanical adaptations that NMT programs can elicit, which may enhance lower limb landing mechanics in young female athletes. The training program used in the current study focused on correction of movement patterns and muscular imbalances with resistance and plyometric training. It is assumed in the literature that NMT improves motor skills of athletes, which can decrease injury risk (Chappell and Limpisvasti, 2008). Myer et al. (2005) found NMT emphasizing deep knee flexion during plyometric and strength exercises significantly improves knee flexion angle during landing, which may decrease injury risk. This is similar to the results found in the current study, whereby the NMT program utilized also placed a large emphasis on deep knee flexion during both the plyometric and strength components of the program, resulting in improved lower extremity biomechanics.

Following maturation it has been reported that post-pubertal female athletes land with greater knee extension than pre-pubertal female athletes (Yu et al., 2002; Hass et al., 2003). This may be partly due to decreased neuromuscular control when landing from a jump commonly seen following maturation in female athletes (Hewett et al., 2004). Additionally, this may also be due to the increase in limb length following maturation providing a greater moment arm length for females to handle, which in the absence of concomitant strength influences them to compensate by landing with the knee in a more extended position. Landing with an extended knee is likely to result in greater ground reaction forces, due to a rapid change in kinetic energy the muscles are in a disadvantageous position to safely absorb landing forces (Podraza and White, 2010). Quatman et al. (2006) showed that as female athletes matured they did not demonstrate any changes in landing forces despite their body mass increasing, thus exposing them to higher relative landing forces. Trained youth netball athletes in the present study were able to significantly decrease peak landing forces following the training intervention, in conjunction with increased knee flexion angle upon landing.

Steele and Milburn (1987) reported average landing forces generated by netball athletes ranged from 3.9 to 4.3 × bodyweight during a typical netball attacking movement pattern which required players to run forward, “break” to a specified side, catch a ball landing on one leg, pivot and throw the ball to a catcher. Landing forces in the present study prior to the training intervention showed youth netball athletes landed with 2.3 × bodyweight in a DVJ task and 1.9 × bodyweight in a SL landing task. These findings are a lot less than the 3.53–5.74 × bodyweight landing forces reported by Otago (2004) for a series of landing patterns more specific to netball. These tasks were more specific netball movement patterns commonly executed in a game which included change of direction and catching or passing a ball. This may indicate landing forces are much greater during the netball attacking movement pattern than in the DVJ or SL landing tasks used in this study. Moreover, it is important to note the EG in the present study were able to largely and significantly decrease their landing force by 1.2 × bodyweight during the box drop and 1.3 × body weight in the vertical jump portion of the DVJ task after completion of the training intervention. Support for these findings can be seen in the work of Hewett et al. (1996) who found that following a plyometric program adolescent female athletes were able to significantly decrease their landing forces by 1.2 × bodyweight following a vertical jump. The ability to reduce landing forces is particularly important for the maturing female athlete due to the relationship between high landing forces and knee injury (Hewett et al., 2006). Therefore, a decrease in landing force may translate to reduced force absorption onto the connective and skeletal structures, decreasing load on the ACL and subsequently ACL injury risk (Myer et al., 2009). The results of the present study show both the combined resistance and plyometric training program were able to produce reductions in landing force in young netball athletes. The current training program emphasized landing with deep knee flexion and to softly absorb landing forces, which may explain the large decrease in GRF following the training intervention.

Knee internal rotation appears to be present in ACL injury situations and usually occurs in combination with valgus knee motion (Koga et al., 2010). Therefore, NMT interventions that are capable of increasing external rotation of the knee have the potential to reduce the amount of stress on the ACL during landing tasks, which may result in a minimisation of knee injury risk (Pfile et al., 2013). The present study found trained participants were able to significantly decrease their knee internal rotation angle following the training intervention. These findings are in agreement with Pfile et al. (2013) who found following a plyometric and core stability program, high school aged female athletes were able to significantly reduce their knee internal rotation angles. The core stability program utilized by Pfile et al. (2013) primarily focused on abdominal exercises, although they did incorporate specific lower body strengthening exercises such as; squats, lunges, hamstring bridges and lumbar extension exercises. Similarly, the strength training component of the present study also included specific lower body resistance exercises executed under load such as; back squats, lunges, split squats and Romanian deadlifts. A meta-analysis by Lesinski et al. (2016) revealed youth athletes respond better to high intensity (80–89% of 1RM) resistance training for improving muscular strength in comparison to lower training intensities (< 79% of 1RM). Therefore, NMT programs that incorporate loaded exercises appear to be important in improving lower extremity biomechanics in youth female athletes. Nevertheless, in order to maintain subsequent strength and performance adaptations, athletes in the current study would ultimately need to extend to higher loads. However, it is important to note that technical competency must be upheld at all times and developed prior to executing high loads. Further, to decrease the imbalance between the hamstring and quadriceps musculature incorporation of exercises that promote hamstring activation and strengthening, such as the Romanian Deadlift as used in the present study are important in protecting and stabilizing the knee joint (Hewett et al., 1999).

The effect of the training program on frontal plane knee abduction motion during landing in the DVJ task demonstrated a large and significant increase in the distance between the two lateral knee markers in the trained participants indicating decreased frontal plane knee abduction motion following the 6-week study period. In contrast, the CG showed a decrease in the distance between the two lateral knee markers during landing. The observed increase in the distance between the right and left lateral knee markers designates that the trained participants were able to decrease their knee abduction motion in the frontal plane after completing the 6 week NMT intervention. This may be due to improved landing mechanics as a result of the NMT intervention. It is widely agreed in the literature that female athletes land with increased knee abduction motion in comparison to males (Ford et al., 2003), this increased knee abduction motion has been shown to be a primary predictor of ACL injury risk due to increased ACL loading (Hewett, 2005). Stuelcken et al. (2012) also found increased knee abduction motion in high performance adolescent netball athletes during a SL landing task. As SL landings are commonly performed in practice and competition with netball athletes landing on one leg approximately 67% of the time (Lavipour, 2011) and the primary cause of lower limb injury being incorrect landing technique combined with rapid deceleration and abrupt landing, the need for NMT programs that reduce abduction knee motion are vital to prevent knee injury in netball athletes. Especially during pre-adolescence (Myer et al., 2009). The present study revealed reductions in abduction knee motion in the sagittal plane during the SL landing task following the intervention in both groups. Although not statistically significant, trained participants made a 2.4 times higher reduction in knee abduction motion then the CG at maximum knee flexion-extension ROM, this change revealed a medium effect size. Therefore, in accordance with previous literature, implementation of NMT programs that alter knee biomechanics and reduce abduction knee motion during the landing phase of a jump appear particularly important in reducing ACL injury risk in youth netball athletes.

Implementation of NMT to reduce ACL injury incidence has been shown to be more effective for athletes under the age of 18 (Yoo et al., 2010; Myer et al., 2013). The findings of Myer et al. (2013) supports the rationale for the current study highlighting the need for effective NMT programs in younger athletes. Following puberty, female athletes demonstrate decreased neuromuscular control of the knee in comparison to males, with this loss of neuromuscular control creating altered landing mechanics placing the knee in a disadvantageous position and thus increasing ACL injury risk (Hewett et al., 2004). Knee extensor strength relative to body mass continues to increase through puberty, while knee flexor strength plateaus. This magnifies the imbalance in ham:quad ratio, which increases quadriceps dominance, thereby placing them at increased risk of ACL injury. Therefore, the NMT interventions are critical with a particular emphasis on posterior chain strength development (Quatman-Yates et al., 2013). A meta-analysis by Myer et al. (2013) demonstrated NMT programs would be best implemented during pre- or early-adolescence prior to the period of altered joint mechanics to decrease knee injury risk. They found that NMT may increase measures evidential of the neuromuscular spurt in females. Although plyometric training is important for injury prevention in young female athletes, a sound resistance training component is also imperative for youth athletes as they show the greatest strength adaptations in response to higher intensities (Lesinski et al., 2016), they also require adequate volume to provide an appropriate adaptive stimulus (Behringer et al., 2010). A meta-analysis by Sugimoto et al. (2014) found greater preventative effects with higher NMT volume inclusive of 2 or more sessions per week, exceeding 30 min, providing a higher benefit for ACL injury risk reduction in high school female athletes. Therefore, it appears that NMT programs which utilize progressive overload as presented in the current study are most beneficial in reducing ACL injury risk factors in youth female athletes in comparison to completing netball training alone.

It should be noted that there are potential limitations with the current study. The inability to control the volume and frequency of netball training between each group may have impacted the results of the study. One of the main goals of the present study was to add resistance training to the netball practice of youth athletes in accordance with best practice recommendations for youth resistance training (Faigenbaum et al., 2016). While it is possible that adding more netball specific training in lieu of strength training might have resulted in improvements in injury risk factors it is important to note that youth sport practice and games may not enable the young athlete to accumulate the recommended amount of moderate-to-vigorous physical activity, as a large proportion of time in practice (and even competition) can be spent in sedentary or low-intensity physical activities (Leek et al., 2011; Guagliano et al., 2013). While the design of the current study is in-line with previous NMT interventions in young athletes (Sander et al., 2013; Keiner et al., 2014), it would be prudent to examine the effects of combined NMT and netball training vs. equated volumes of solely netball training. Although the present study was not designed to answer this specific question it is important to contextualize the fact that the present data suggests that simply adding a NMT program to regular netball training activities of youth netball players does in fact result in significant training benefits. These benefits are noted in the two landing tasks presented in the present study. It is however possible that more complex landing tasks that incorporate landing as well as sport specific skill such as catching and throwing need to be evaluated to develop a more comprehensive understanding of the impact of a NMT program in youth athletes. Even though the present study was not designed to specifically determine the neuromuscular factors that underpin the noted differences in landing mechanics, it does suggest that a NMT confers benefits to the youth netball player. Future research is warranted in order to understand the neuromuscular factors as well as to determine the long-term impact of integrating a NMT program in the preparation of youth Netball Players.

## Conclusion

Netball has a reputation for a high incidence of knee injuries, with most injuries occurring during abrupt landings from a jump combined with incorrect landing technique. Therefore, it appears important that implementation of NMT programs that teach youth netball athletes proper landing technique and to safely absorb landing forces are important in the prevention of ACL injuries. The results of this study provide evidence that NMT programs encompassing progressive resistance and plyometric training decrease associated risk factors for non-contact ACL injuryincluding poor knee-joint biomechanics and increased landing forces in youth female netball athletes. Much of the benefit of the program appeared to be through improved knee flexion-extension ROM, increased knee external rotation angle, decreased frontal plane knee abduction motion, and decreased landing forces. It is important to note that the current study used athletes who had no history of strength training prior to participating in the present study. As such for ongoing strength and performance adaptations to occur this program should be contextualized as part of a long-term athlete development program which when appropriately applied results in long term physical adaptations that are above and beyond that of growth and maturation.

## Author contributions

AH: worked on study design, data collection, data analysis, and manuscript preparation. EH: assisted with training intervention design, delivery of training, data collection, and manuscript preparation. CJ: assisted with study design, worked on all biomechanical analyses, and assisted with manuscript preparation. RL: assisted with study design, data analysis and manuscript preparation. GH: assisted with study design, data collection, data analysis, and manuscript preparation.

### Conflict of interest statement

The authors declare that the research was conducted in the absence of any commercial or financial relationships that could be construed as a potential conflict of interest.
